# Clinical Phenotype Comparison in Polish Patient Cohorts with and Without Molecular Diagnosis of Dystonia

**DOI:** 10.3390/jcm15103975

**Published:** 2026-05-21

**Authors:** Lukasz Milanowski, Marta Jurek, Anna Salińska, Aleksandra Podwysocka, Monika Figura, Stanisław Szlufik, Maciej Geremek, Julia Nowak, Krzysztof Szczałuba, Dorota Hoffman-Zacharska, Dariusz Koziorowski

**Affiliations:** 1 Department of Neurology, Faculty of Health Science, Medical University of Warsaw, 03-242 Warsaw, Poland; lukasz.milanowski@wum.edu.pl (L.M.); salinska.anna@gmail.com (A.S.); monika.figura@wum.edu.pl (M.F.); stanislaw.szlufik@wum.edu.pl (S.S.); julia.nowak@wum.edu.pl (J.N.); 2 Department of Medical Genetics, Institute of Mother and Child, 01-211 Warsaw, Poland; marta.jurek@imid.med.pl (M.J.); aleksandra.podwysocka@imid.med.pl (A.P.); maciej.geremek@imid.med.pl (M.G.); dorota.hoffman@imid.med.pl (D.H.-Z.); 3 Department of Medical Genetics, Medical University of Warsaw, 02-106 Warsaw, Poland; krzysztof.szczaluba@wum.edu.pl; 4 Institute of Genetics and Biotechnology, Faculty of Biology, Warsaw University, 02-106 Warsaw, Poland

**Keywords:** dystonia, Polish population, genetics, movement disorders

## Abstract

**Background**: Dystonia is a heterogeneous hyperkinetic movement disorder characterized by sustained or intermittent muscle contractions causing abnormal movements and postures. Although numerous genes associated with dystonia have been identified, the genetic background remains unknown in many patients. Data on genotype–phenotype correlations in Polish populations remain limited. **Objective**: To analyze the clinical characteristics of patients with generalized dystonia and compare clinical features between individuals with and without genetically confirmed dystonia-causative variants in a Polish cohort. **Methods**: A retrospective analysis of patients diagnosed with generalized dystonia at a single neurological center was performed. Diagnosis was established according to MDS criteria. Genetic analysis included whole-exome sequencing, targeted NGS genetic panel, MLPA, Sanger sequencing and PCR_RFLP analysis. Clinical and demographic data were extracted from medical records. Clinical characteristics of individuals with and without causative variants were compared. **Results**: A total of 113 patients with generalized dystonia were included. Genetic variants were identified in 13 patients (11.5%). These included variants within the *TOR1A*, *THAP1*, *SGCE*, *GCH1*, *NKX2-1*, *SLC2A1*, *KMT2B*, *PDHA1*, *MFN2*, and *GNAL* genes. We found detailed clinical data of 46 patients included in the study. Our comparative analysis of patients with causative (*n* = 7) and without causative variants (*n* = 39) revealed no statistically significant differences in age of onset, initial symptom localization, treatment response, family history, or associated neurological features. **Conclusions**: In this cohort of Polish patients with generalized dystonia, we identified pathogenic variants in approximately 11.5% of cases. No significant clinical differences were observed between patients with genetically confirmed dystonia and those without identified variants. In this study, we report the first two Polish cases with DYT-*GNAL* variants. Further studies are required to reveal the clinical heterogeneity of dystonia and characterize dystonia subtypes.

## 1. Introduction

Dystonia can be characterized as both a symptom that is part of a broader clinical picture of certain neurological conditions, and a disease in itself—a hyperkinetic movement disorder in which sustained or repetitive muscle contractions occur involuntarily. A revised classification of dystonia categorizes this condition based on several features, such as body distribution, age at onset, progression pattern, and accompanying symptoms [1]. Body distribution can be focal, segmental, or generalized. Focal dystonia is defined as symptoms limited to one body area. Segmental dystonia manifests in two neighboring regions, whereas generalized dystonia affects the whole body [1,2,3]. Nowadays, novel genetic techniques are applied to discover new genetic factors associated with dystonia occurrence [4]. A genetic background is usually identified in generalized dystonia, particularly in cases with symptom onset before 18 years of age and a positive family history [5]. If the only symptoms are dystonia, the condition is classified as isolated dystonia, whereas if other comorbid symptoms are present, it is referred to as combined dystonia [1,2].

Recently, thanks to the ongoing advancement of molecular genetics’, numerous causative variants have been identified. Approximately 400 have been associated with dystonia According to the OMIM database, 38 genes are related to monogenic forms (OMIM; Phenotypic Series—PS128100, omim.org/phenotypicSeries/PS128100); however, in most cases, a genetic underlying cause remains unidentified. As in some cases, the response to certain treatments may vary depending on the patients ‘genetic status [6]. Therefore, there is a strong need to determine this status in new populations. There is limited data on cross-sectional cohort analyses in Europe. However, we found several reports from large cohort studies. A German paper included a large cohort from their national DysTract registry, which revealed 137 distinct likely pathogenic/pathogenic variants. The authors concluded that generalized dystonia and an age of onset <30 years old were the strongest predictors of an underlying genetic basis of the disease [7]. Other studies focused on the specific type of dystonia or gene responsible for the disease, such as the *GCH1* variant in the Spanish population or cervical dystonia and blepharospasm in the Hungarian population [8,9]. To date, studies including the Polish population were mostly limited to case reports or single gene analyses, which highlights the need for future larger cohort investigations [10,11,12,13,14,15,16].

The aim of this study was to conduct a single-center analysis of patients with generalized dystonia and correlate their clinical symptoms with whether they possessed known variants in genes associated with dystonia.

## 2. Materials and Methods

### 2.1. Clinical Analysis

The MDS diagnostic criteria were applied by experienced movement disorder specialists for the diagnosis of generalized dystonia (D.K., M.F., S.S. and L.M.) [1,2]. Clinical and demographic data were collected retrospectively using past medical records from the Department of Neurology, Faculty of Health Science, Medical University of Warsaw. The study received approval from the Bioethics Committee of the Medical University of Warsaw and was conducted in accordance with the ethical principles outlined in the Declaration of Helsinki (KB/56/2018). Most of the patients were initially screened for DYT-*TOR1A* variants, as this represents the most common genetic cause of generalized dystonia [17]. Further targeted genetic analyses were performed when specific clinical features were present, for example, myoclonus suggestive of an SGCE variants or thyroid dysfunction suggestive of an NKX2-1 variants.

### 2.2. Genetic Analysis

Genetic analysis was performed in all patients included in the study. Molecular analyses were performed on gDNA isolated from peripheral blood leukocytes using standard procedures. Different molecular techniques were implemented, depending on clinical referral (Supplementary Table S1).

Different molecular techniques were implemented, depending on clinical referral.

In regard to the most common *DYT1*, the analysis included the presence of ΔGAG *DYT1* deletion in the *TOR1A* gene using polymerase chain reaction–restriction fragment length polymorphism (PCR-RFLP). This analysis was performed in 103 patients. Sanger sequencing as a singular method was used in 44 patients. It included the following genes: *GCH1* (2 patients), *THAP1* (28 patients), *SGCE* (17 patients), *SLC2A1* (8 patients), *PNKD* (2 patients), *PRRT2* (4 patients) *NKX2-1* (1 patient), *TH* (1 patient) and *SPR* (1 patient). MLPA as a singular method was performed in 23 patients. The MRC Holland Salsa P099, P059 and P138 (Amsterdam, The Netherlands) was used for analysis, including *GCH1*, *TH*, *SGCE*, *PRRT2* (P099; 16 patients), *TOR1A*, *THAP1*, *ATP1A3*, and *PRKRA* (P059; 9 patients), *SLC2A1* and *STXBP1* (P138; 4 patients) gene exon dosage probes.

The targeted NGS sequencing panel was performed in 4 patients. The panel which included the following genes was analyzed: *ADAR*, *ADCY5*, *ANO3*, *ATM*, *ATP1A3*, *ATP7B*, *BCAP31*, *CACNA1A*, *COASY*, *COL6A3*, *COX20*, *DNAJC12*, *FA2H*, *FBXO7*, *FTL*, *GCDH*, *GCH1*, *GNAL*, *HPCA*, *KCD17*, *KCNA1*, *KCNMA1*, *KIF1C*, *KMT2B*, *MECR*, *PANK2*, *PINK1*, *PLA2G6*, *PNKD*, *PRKN*, *PRKRA*, *PRRT2*, *SCN8A*, *SGCE*, *SLC19A3*, *SLC2A1*, *SLC30A10*, *SLC39A14*, *SLC6A3*, *SPR*, *TAF1*, *TH*, *THAP1*, *TOR1A*, *TUBB4A*, *VAC14*, *VPS13A*. Targeted genes were enriched using KAPA Hyper Choice and sequencing was then performed using the MiSeq platform (Illumina, San Diego, CA, USA).

WES was performed in 2 patients. The DNA library was prepared using the Human Exome 2.0 Plus Comprehensive Exome Spike-in (TwistBioScience, South San Francisco, CA, USA) and sequencing was performed on NovaSeq6000 platform (Illumina, USA).

Sequences were mapped and compared to the human reference genome version GRCh38/hg38.

Sequencing data were analyzed using a robust end-to-end in-house pipeline “IMC_pipeline” (data on request), with annotations of variants VEP (Ensembl release 114).

The presence of all variants identified by NGS was later confirmed with target Sanger sequencing.

The identified variants were referred to canonical reference sequences GRCh38 NM_ MANE Select [18] and described according to HGVS v.21.0.4 recommendations [19].

The most identified variants have been previously documented in the Human Gene Mutation Database Professional [20] and/or ClinVar database [21].

The pathogenicity of identified variants was classified according to American College of Medical Genetics and Genomics standards and guidelines [22].

### 2.3. Statistical Analysis

The statistical analyses were conducted using STATISTICA v.13.5 software, TIBCO, Palo Alto, CA, USA. The normality of distribution was evaluated with the Shapiro–Wilk test. Continuous variables were presented as means and standard deviations. Categorical variables were presented as frequencies (percentages). Parametric data were compared using the independent *t*-test, and the categorical data were compared with Fisher’s exact test (two-sided).

## 3. Results

A total number of 113 patients (44.2% males (*n* = 50)) were included in the analysis, which revealed 13 with causative variants (11.5%)-3 *TOR1A* (2.7%), 1 *THAP1* (0.9%), 1 *SLC2A1* (0.9%), 1 *NKX2*-1 (0.9%), 1 *GCH1* (0.9%), 2 *SGCE* (1.8%), 2 *GNAL1* (1.8%), 1 *PDHA1* (0.9%) and 1 *MFN2* (0.9%) in 1 patient and 1 *KMT2B* (0.9%) (Table 1). Due to the retrospective nature of the study, only 46 patients possessed detailed clinical data, 7 of which had confirmed causative variants. The age of onset was similar in patients with and without causative variants. There were no differences in the body region of first symptom onset. There were no differences in the response to any treatment. The detailed results can be found in Table 2.

## 4. Discussion

Generalized dystonia remains one of the most disabling and burdensome movement disorder [23]. Identifying the genetic background is clinically important, as some forms of dystonia may have different responses to various therapeutic approaches depending on the underlying variant. For example, DYT-*KMT2B* and DYT-*TOR1A* dystonias have been reported to respond particularly well to deep brain stimulation (DBS), showing the importance of genetic diagnoses in clinical decision-making [24,25]. In our cohort, we found one patient with *KMT2B* and three patients with DYT-*TOR1A*. One female DYT-*TOR1A* subject responded very well to bilateral DBS GPi.

In our cohort, we cautiously observed that establishing a specific genetic diagnosis based solely on clinical symptoms of generalized dystonia may be difficult. Although some distinct clinical features may be associated with specific genetic forms, their clinical presentation is often highly heterogeneous. For example, thyroid dysfunction and pulmonary involvement have been reported in *NKX2-1*-related dystonia, whereas *GCH1*-related dystonia typically presents before the age of 18 years with lower limb dystonia and a good response to levodopa treatment [26,27,28]. Additionally, some forms of dystonia associated with neurodegeneration related to brain iron accumulation or Wilson disease may present with characteristic neuroimaging features [29].

Our study analyzed a population that has been underrepresented in genetic studies of dystonia. The most interesting findings in our report are two cases possessing the *GNAL* variant. Interestingly it was responsible for almost 2% of all generalized dystonia in our cohort. In the first family, one patient presented with symptoms involving the neck and limbs, with symptom onset at the age of 14. The p.Glu129Lys variant was not previously reported and had no functional studies. However, it has a high CADD score could be potentially responsible for the patient’s dystonia. The second family possessed the heterozygous *GNAL* deletion with a concomitant *AFGL32* deletion reported in the proband. The symptoms initially began in the face and neck at 18 years of age. The patient underwent DBS GPI at 37 years of age with no clinical response. Notably, the patient had no significant family history. However, the NGS panel study is not a method of choice to confirm the copy number variants and requires additional confirmation in methods like MLPA or aCGH [30].

*GNAL* variants have previously been associated with primary dystonia and almost 100 patients have been reported to date [31,32]. The inheritance pattern is typically autosomal dominant with limited penetrance in affected families [33,34]. Symptom onset usually occurs from early to late adulthood, with a median age of onset of around 38 years. In most patients, symptoms begin in the neck and craniocervical region and may progress to segmental, multifocal, or occasionally generalized dystonia. The *GNAL* gene is located on the short arm of chromosome 18 (18p11.21) Additional clinical features can include spinocerebellar ataxia, facio-scapulo-humeral muscular dystrophy, and dystonia, with *GNAL* deletion considered the main driver of dystonia in this syndrome. Treatment is usually limited, but there have been reports of 15 cases of individuals who underwent DBS surgery, mostly revealing improvement [32]. The second patient has a concomitant deletion of the *AFGL32* gene. The variant in this gene is associated with several diseases like spinocerebellar ataxia type 28, optic nerve atrophy, spastic ataxia type 5 and dystonia parkinsonism. *AFG3L2* interacts with *OPA1*, which encodes a mitochondrial GTPase responsible for the fusion of the inner mitochondrial membrane [35].

*SLC2A1* variants are associated with glucose transporter type 1 deficiency syndrome (GLUT1-DS). Seizures are the most reported symptom, followed by movement disorders such as paroxysmal exertional dyskinesia, dystonia, and ataxia. A ketogenic diet is the first-line treatment [36]. A heterozygous *SLC2A1* p.Arg218Cys variant was identified in a patient whose symptoms began at 22 years of age. The initial presentation was cervical dystonia, which later progressed to more generalized symptoms. A ketogenic diet was recommended; however, due to loss to follow-up, the treatment outcome remains unknown.

We also identified two new patients with *SGCE* variants in our cohort, which is the most common genetic cause of myoclonus-dystonia. In Poland, the *SGCE* variants has previously been reported in two families [14,15].

We also found a patient with levodopa-responsive dystonia. Variants in *GCH1* are responsible for dopa-responsive dystonia and are typically associated with early onset and a good response to levodopa treatment [36]. In Poland, only three cases of *GCH1* variants have been previously reported. Interestingly, the variant identified in our study had also been reported in a 15-year-old Italian patient presenting with gait disturbances [37].

We also included into our study three patients previously reported on in the literature with DYT-*TOR1A* variants [13]. DYT-*TOR1A* was the first genetically described form of dystonia and typically symptoms manifest before the age of 18 [38]. Previous studies estimated the prevalence of the *TOR1A* variant in patients with dystonia to be approximately 6% [39]. However, only two previous studies have analyzed *TOR1A* variants in Polish patients with dystonia. Those reports showed a relatively high prevalence (20.8% and 7%), which is higher than that observed in our cohort (2.7%) [11,13]. This discrepancy may be associated with smaller sample sizes in previous studies as well as differences in patient selection. In addition, our cohort only included patients with generalized dystonia.

The previously reported patient had two confirmed variants: an X-linked PDHA1 variant (causing pyruvate dehydrogenase complex deficiency) and an autosomal dominant MFN2 variant responsible for Charcot–Marie–Tooth disease type 2A2A or hereditary motor and sensory neuropathy type VIA16. The patient’s first symptoms appeared at 8 years of age, which is unusual for pyruvate dehydrogenase complex deficiency, as the disease typically presents in infancy and only a few patients survive into late childhood. The coexistence of the second mutation may have contributed to the unique phenotype of generalized dystonia [16]. Patients with confirmed variants included in this study were also reported in other studies: *NKX2-1* [26,40], *PDHA1* and *MFN2* [16], *THAP1* [12].

Several limitations of this study should be acknowledged. Firstly, the retrospective design resulted in incomplete clinical data for some patients, and genetic testing approaches were not uniform across the cohort. Additionally, certain variants identified through NGS-based panels—such as copy number variations (CNVs) and exonic rearrangements—require confirmation using complementary methods, including array comparative genomic hybridization. The pathogenicity of variants of uncertain significance require further investigation in a functional study setting. Moreover, the relatively small sample size in the comparative analysis limits the statistical power of the study. As a result, the lack of statistically significant differences between patients with and without causative variants should be interpreted with caution. Despite these limitations, this study represents the first analysis of this kind in the Polish population. Future research involving larger cohorts and more comprehensive genetic testing strategies may enable more precise genotype–phenotype correlations.

## 5. Conclusions

Our study presents an overview of the genetic background of generalized dystonia in a Polish cohort. Pathogenic variants were identified in several dystonia-associated genes; however, no clear differences in clinical presentation were observed between patients with and without dystonia causative variants. These findings underscore the clinical heterogeneity of generalized dystonia and highlight the importance of genetic testing for accurate diagnosis. Furthermore, genetic analysis should be routinely considered in patients with generalized dystonia, as clinical features alone may not reliably predict the underlying etiology.

## Figures and Tables

**Table 1:** Table with variants discovered in analyzed cohort.

Patient	Sex	Gene Name	Variant		CADD Phred	Mode of Inheritance	Zygosity	Classification	Previously Reported	Reference Sequences
			c.DNA	Protein						
P1	female	*TOR1A*	c.907_909delGAG	p.Glu303del	19.72	AD	heterozygous	P	Yes/ClinVar RCV000005488.8; HGMD 2026.1 CD972174	NM_000113.3
P2	female	*TOR1A*	c.907_909delGAG	p.Glu303del	19.72	AD	heterozygous	P	Yes/ClinVar RCV000005488.8 HGMD 2026.1 CD972174	NM_000113.3
P3	female	*TOR1A*	c.907_909delGAG	p.Glu303del	19.72	AD	heterozygous	P	Yes/ClinVar RCV000005488.8 HGMD 2026.1 CD972174	NM_000113.3
P4	male	*THAP1*	c.15C>G	p.Cys5Trp	23.40	AD	heterozygous	LP	Yes/VCV000807708.4 HGMD CM149026	NM_018105.3
P5	male	*NKX2*-1	c.348del	Cys117Alafs*8	-	AD	heterozygous	LP	No	NM_001079668.3
P6	female	*SGCE*	c.1310C>T	p.Pro437Leu	26.80	AD	heterozygous	VUS	Yes/ClinVar VCV000373239.5	NM_003919.3
P7	female	*SGCE*	c.742T>C	p.Cys248Arg	27.70	AD	heterozygous	LP	No	NM_003919.3
P8	male	*SLC2A1*	c.652C>T	p.Arg218Cys	25.70	AD	heterozygous	VUS	Yes/ClinVar VCV000095415.19; HGMD 2026.1 CM2318414	NM_006516.4
P9	female	*GNAL*	c.385 G>A	p.Glu129Lys	35.00	AD	heterozygous	VUS	No	NM_001142339.3
P10	female	*GNAL*	ex1-12del ^#^	p.?		AD	heterozygous	P	Yes/ClinVar VCV000148429.2, VCV000154072.3, VCV002579191.1; HGMD 2026.1 CG177362, CG1413517, CG2312382	NM_001142339.3
P10	female	*AFG3L2*	ex10-17del ^##^	p.?		AD	heterozygous	P		
P11	female	*GCH1*	c.453+1G>A	p.?	32.00	AD	heterozygous	P	Yes/ClinVar VCV002925519.3; HGMD 2026.1 CS093875	NM_000161.3
P12	male	*MFN2*	c.2119C>T	p. Arg707Trp	29.30	AD	heterozygous	P/LP	Yes/ClinVar VCV000002280.80; HGMD 2026.1 CM081695	NM_014874.4
P12	male	*PDHA1*	c.784 G>C	p.Val262Leu	25.50	XLD	hemizygous	P	Yes/ClinVar VCV004070370.1; HGMD 2026.1 CM2213231	NM_000284.4
P13	female	*KMT2B*	c.2885G>A	p.Arg962His	28.90	AD	heterozygous	LP	Yes/ClinVar VCV002498773.24	NM_014727.3

^#^ NC_000018.10:g.[(?_11689564)_(11881135_?)del];[=], ^##^ NC_000018.10:g.[(?_12377082)_(12329565 _?)del];[=], AD—autosomal dominant, LP—likely pathogenic, P—pathogenic, XLD—X-linked dominant.

**Table 2:** Comparison of the clinical data of patients withs (*n* = 7) and without (*n* = 39) variants associated with dystonia.

	Patients with Dystonia-Associated Variants (*n* = 7)	Patients Without Dystonia-Associated Variants (*n* = 39)	*p* Value
Age of onset	19.0 ± 12.7	25.4 ± 24.2	0.40
First symptom: lower limb onset	0	13 (27.3%)	0.16
First symptom: trunk onset	1 (14.2%)	7 (16.73%)	1
First symptom: upper limb onset	2 (28.6%)	25 (64.1%)	0.09
First symptom: head and neck onset	3 (42.9%)	25 (64.1%)	1
Positive family history	3 (42.9%)	14 (35.9%)	0.65
Response to any treatment	1 (14.2%)	23 (69.7%)	0.06
Myoclonus presence	2 (28.6%)	8 (20.5%)	0.63
MRI changes	3 (42.9%)	13 (33.3%)	0.4
Chorea presence	1 (14.2%)	1 (2.6%)	0.29
Thyroid insufficiency	2 (28.6%)	1 (2.6%)	0.28

## Data Availability

The data presented in this study are available on request from the corresponding author due to ethical reasons.

## Supplementary Materials

The following supporting information can be downloaded at https://www.mdpi.com/article/10.3390/jcm15103975/s1, Table S1: Different molecular techniques implementation.

## Abbreviations

Table following abbreviations are used in this manuscript:

ACMG	American College of Medical Genetics and Genomics
AD	Autosomal dominant
ADCY5	Adenylate cyclase 5
ADAR	Adenosine deaminase RNA specific
AFG3L2	AFG3 like matrix AAA peptidase subunit 2
aCGH	Array comparative genomic hybridization
ANO3	Anoctamin 3
ATM	ATM serine/threonine kinase
ATP1A3	ATPase Na+/K+ transporting subunit alpha 3
ATP7B	ATPase copper transporting beta
BCAP31	B-cell receptor associated protein 31
CACNA1A	Calcium voltage-gated channel subunit alpha 1A
CADD	Combined Annotation Dependent Depletion
CNV/CNVs	Copy number variation/copy number variations
COASY	Coenzyme A synthase
COL6A3	Collagen type VI alpha 3 chain
COX20	Cytochrome c oxidase assembly factor COX20
DBS	Deep brain stimulation
DNA	Deoxyribonucleic acid
DNAJC12	DnaJ heat shock protein family member C12
DRD	Dopa-responsive dystonia
DYT	Dystonia
DYT-GNAL	GNAL-related dystonia
DYT-KMT2B	KMT2B-related dystonia
DYT-SGCE	SGCE-related myoclonus-dystonia
DYT-THAP1	THAP1-related dystonia
DYT-TOR1A	TOR1A-related dystonia
DYT1	Dystonia type 1
FA2H	Fatty acid 2-hydroxylase
FBXO7	F-box protein 7
FSHD	Facioscapulohumeral muscular dystrophy
FTL	Ferritin light chain
GCDH	Glutaryl-CoA dehydrogenase
GCH1	GTP cyclohydrolase 1
gDNA	Genomic DNA
GLUT1-DS	Glucose transporter type 1 deficiency syndrome
GNAL	G protein subunit alpha L
GPi	Globus pallidus internus
GRCh38/hg38	Genome Reference Consortium Human Build 38
GTP	Guanosine triphosphate
GTPase	Guanosine triphosphatase
HGMD	Human Gene Mutation Database
HGVS	Human Genome Variation Society
HPCA	Hippocalcin
KCNMA1	Potassium calcium-activated channel subfamily M alpha 1
KCNA1	Potassium voltage-gated channel subfamily A member 1
KCTD17	Potassium channel tetramerization domain containing 17
KIF1C	Kinesin family member 1C
KMT2B	Lysine methyltransferase 2B
MANE	Matched Annotation from NCBI and EMBL-EBI
MDS	Movement Disorder Society
MECR	Mitochondrial trans-2-enoyl-CoA reductase
MFN2	Mitofusin 2
MLPA	Multiplex ligation-dependent probe amplification
NGS	Next-generation sequencing
NKX2-1	NK2 homeobox 1
OMIM	Online Mendelian Inheritance in Man
OPA1	OPA1 mitochondrial dynamin-like GTPase
PANK2	Pantothenate kinase 2
PCR-RFLP	Polymerase chain reaction–restriction fragment length polymorphism
PDHA1	Pyruvate dehydrogenase E1 subunit alpha 1
PED	Paroxysmal exertional dyskinesia
PINK1	PTEN induced kinase 1
PLA2G6	Phospholipase A2 group VI
PNKD	Paroxysmal non-kinesigenic dyskinesia
PRKN	Parkin RBR E3 ubiquitin protein ligase
PRKRA	Protein activator of interferon-induced protein kinase EIF2AK2
PRRT2	Proline rich transmembrane protein 2
SCA28	Spinocerebellar ataxia type 28
SCN8A	Sodium voltage-gated channel alpha subunit 8
SGCE	Sarcoglycan epsilon
SLC19A3	Solute carrier family 19 member 3
SLC2A1	Solute carrier family 2 member 1
SLC30A10	Solute carrier family 30 member 10
SLC39A14	Solute carrier family 39 member 14
SLC6A3	Solute carrier family 6 member 3
SPR	Sepiapterin reductase
STXBP1	Syntaxin binding protein 1
TAF1	TATA-box binding protein associated factor 1
TH	Tyrosine hydroxylase
THAP1	Thanatos-associated domain containing 1
TOR1A	Torsin family 1 member A
TUBB4A	Tubulin beta 4A class IVa
VAC14	VAC14 component of PIKFYVE complex
VEP	Variant Effect Predictor
VPS13A	Vacuolar protein sorting 13 homolog A
VUS	Variant of uncertain significance
WES	Whole-exome sequencing
